# RL-QPSO net: deep reinforcement learning-enhanced QPSO for efficient mobile robot path planning

**DOI:** 10.3389/fnbot.2024.1464572

**Published:** 2025-01-08

**Authors:** Yang Jing, Li Weiya

**Affiliations:** Hebi Institute of Engineering and Technology, Henan Polytechnic University, Hebi, Henan, China

**Keywords:** path planning, Quantum-behaved Particle Swarm Optimization, deep reinforcement learning, mobile robotics, complex environments

## Abstract

**Introduction:**

Path planning in complex and dynamic environments poses a significant challenge in the field of mobile robotics. Traditional path planning methods such as genetic algorithms, Dijkstra's algorithm, and Floyd's algorithm typically rely on deterministic search strategies, which can lead to local optima and lack global search capabilities in dynamic settings. These methods have high computational costs and are not efficient for real-time applications.

**Methods:**

To address these issues, this paper presents a Quantum-behaved Particle Swarm Optimization model enhanced by deep reinforcement learning (RL-QPSO Net) aimed at improving global optimality and adaptability in path planning. The RL-QPSO Net combines quantum-inspired particle swarm optimization (QPSO) and deep reinforcement learning (DRL) modules through a dual control mechanism to achieve path optimization and environmental adaptation. The QPSO module is responsible for global path optimization, using quantum mechanics to avoid local optima, while the DRL module adjusts strategies in real-time based on environmental feedback, thus enhancing decision-making capabilities in complex high-dimensional scenarios.

**Results and discussion:**

Experiments were conducted on multiple datasets, including Cityscapes, NYU Depth V2, Mapillary Vistas, and ApolloScape, and the results showed that RL-QPSO Net outperforms traditional methods in terms of accuracy, computational efficiency, and model complexity. This method demonstrated significant improvements in accuracy and computational efficiency, providing an effective path planning solution for real-time applications in complex environments for mobile robots. In the future, this method could be further extended to resource-limited environments to achieve broader practical applications.

## 1 Introduction

Robot path planning is an important research direction in the fields of robot navigation and automation control (Liu et al., 2023). It has wide applications in industrial automation, such as automated production lines and warehouse logistics systems, as well as in various domains like intelligent transportation, autonomous driving, and home service robots, showcasing significant potential. Research in robot path planning not only improves the accuracy and efficiency of robot autonomous navigation but also enhances the robot's adaptability to the environment and task execution capabilities (Sanchez-Ibanez et al., 2021). The rapid advancement of artificial intelligence and sensing technologies has led to the optimization and innovation of path planning algorithms. This not only enhances robot technology but also drives technological advancements in related application fields (Yang et al., 2020). Therefore, in-depth research and addressing key issues in robot path planning hold substantial practical significance in driving the overall development of robotics technology.

In the early days of robotics, path planning primarily relied on basic graph search algorithms. Dijkstra's algorithm (Luo et al., 2020), initially employed for path planning, can find the shortest path from a single source point to all other nodes, ensuring result accuracy. However, its downside is the high computational cost when dealing with large-scale networks. The Bellman-Ford algorithm addresses shortest path problems in graphs with negative edge weights, expanding application scenarios but still lacking in efficiency (Schambers et al., 2018). The Floyd-Warshall algorithm provides a method to compute the shortest paths between all vertex pairs in a graph, ideal for scenarios that require frequent shortest path queries, yet its *O*(*n*^3^) time complexity restricts its application in large graphs (Aziz et al., 2017). The A* algorithm introduces heuristic evaluation to optimize path search, significantly enhancing search efficiency, but its performance heavily depends on the choice of heuristic function Guruji et al. (2016). The IDA* algorithm (Iterative Deepening A*) (Guo et al., 2022) aims to resolve the space limitation issues of A*, adopting a depth-first search approach that incrementally increases cost limits to find paths, reducing memory usage but adding computational complexity.

To address the limitations of traditional graph search algorithms in complex environments, researchers have developed heuristic and metaheuristic approaches that provide improved flexibility and adaptability. The Rapidly-exploring Random Tree (RRT) algorithm (Muis, 2019), for instance, is particularly effective in high-dimensional and unstructured spaces due to its random exploration, which helps avoid local optima. However, RRT's inherent randomness may lead to suboptimal paths and fluctuating computational efficiency (Kuffner and LaValle, 2000). Similarly, the Probabilistic Roadmap (PRM) (Latombe, 1998) uses random sampling in continuous spaces to connect points, making it suitable for relatively stable environments, though it struggles in highly dynamic scenarios. Genetic algorithms (Aybars, 2008), inspired by natural selection, improve solution diversity but often face slow convergence and risk getting trapped in local optima. Simulated annealing introduces a probabilistic mechanism to escape local minima, though its efficiency heavily depends on the design of the cooling schedule. Particle Swarm Optimization (PSO) (Yu et al., 2022), which models social behaviors like flocking, is simple to implement and effective in exploring the search space, but in complex environments, it may require extensive iterations to achieve satisfactory results.

To further improve performance, end-to-end learning-based approaches have emerged, leveraging deep learning techniques to directly map input data (e.g., sensor or image data) to output actions or paths (Riviere et al., 2020). These methods, trained on large datasets, can autonomously learn complex patterns in various environments without relying on predefined heuristics or manually designed features. Convolutional Neural Networks (CNNs) (Wang et al., 2020) and Recurrent Neural Networks (RNNs) (Nair and Supriya, 2020) are often integrated in end-to-end frameworks to handle spatial and temporal information, respectively, allowing for efficient path planning in dynamic scenarios. Although end-to-end methods eliminate the need for intermediate feature extraction and manual tuning, they are computationally intensive and require substantial training data. Nonetheless, end-to-end models provide an adaptive (Teng et al., 2023), flexible approach suitable for real-world applications, as they can continuously improve performance with more data and updates, making them particularly valuable for navigating complex, unpredictable environments.

As computational power has increased and data availability has improved, deep learning technology, particularly deep reinforcement learning, has been widely introduced into path planning to adapt to complex and dynamic environments. Deep Q-Networks (DQN) (Li et al., 2022) combine deep learning with Q-learning, enabling robots to learn effective navigation strategies in complex environments. Although DQN has improved learning performance, it relies on a large amount of interaction data, requires long training periods, and is prone to overfitting. Policy gradient methods (Zhang et al., 2020) enhance learning efficiency by optimizing the policy itself, offering flexible control and allowing the model to learn complex strategies. However, their main drawback is high variance during training, which can lead to unstable learning. Double DQN (Xiaofei et al., 2022) uses two networks to reduce estimation bias, improving algorithm stability but increasing computational complexity and resource demands. Asynchronous Advantage Actor-Critic (A3C) (Leng et al., 2022) accelerates the process and enhances robustness through multi-threaded learning, but its high parallelism requirements may limit its application in resource-constrained environments. Monte Carlo Tree Search (MCTS) (Qian et al., 2022), successfully applied in AlphaGo, selects the optimal strategy by simulating future action sequences but faces challenges of high computational costs and substantial resource demands. Soft Actor-Critic (SAC) (Tang et al., 2023) and Twin Delayed DQN (TD3) (Zhou et al., 2024) provide new directions for deep reinforcement learning. SAC balances policy performance (He et al., 2022) and exploration through the entropy maximization principle, while TD3 reduces overestimation and noise with two value functions. These methods optimize path planning in complex environments but face challenges with complex parameter tuning and algorithm implementation (Garg et al., 2024). Although these methods hold great potential for applications like autonomous driving and drone navigation, the resource consumption and algorithm stability in practical applications need further research and optimization.

Compared to the limitations of traditional and enhanced deep learning methods, this paper introduces an innovative path planning approach–RL-QPSO Net–designed to enhance robot performance in complex dynamic environments. RL-QPSO Net combines Quantum-behaved Particle Swarm Optimization (QPSO) and Deep Reinforcement Learning (DRL), offering a novel solution for path planning tasks. The QPSO module incorporates quantum behaviors to enhance the search capabilities of the swarm, enabling effective exploration of the global optimal path in high-dimensional complex scenarios and avoiding the local optima typical of traditional algorithms. Simultaneously, the deep reinforcement learning module adjusts the robot's path selection strategies through real-time interaction with the environment, granting the model adaptability to dynamically alter its course as the environment changes. By organically integrating these two modules, RL-QPSO Net not only achieves robustness optimization across various environments but also significantly enhances the model's global convergence and path planning accuracy. Experimental results demonstrate that RL-QPSO Net outperforms traditional methods on multiple datasets, showing significant advantages in accuracy, efficiency, and adaptability, thus providing an efficient and stable solution for mobile robot path planning tasks.

- A novel path planning approach, RL-QPSO Net, is introduced, combining Quantum-behaved Particle Swarm Optimization (QPSO) with Deep Reinforcement Learning (DRL) modules, innovatively enhancing the global optimality capabilities of path planning.
- This method exhibits high efficiency and versatility across multiple scenarios, with the QPSO module enhancing search capabilities in high-dimensional complex environments and the DRL module ensuring real-time adaptability in dynamic settings.
- Experimental results demonstrate that RL-QPSO Net significantly outperforms traditional methods across multiple datasets, showing distinct advantages in accuracy, computational efficiency, and adaptability, making it suitable for practical applications in complex environments.

## 2 Methodology

### 2.1 Overview of our network

In our proposed RL-QPSO Net model, we introduce a novel architecture that leverages deep reinforcement learning (DRL) coupled with Quantum-behaved Particle Swarm Optimization (QPSO) to address the complex and dynamic nature of mobile robot path planning. The model is designed to efficiently navigate through unpredictable environments by integrating adaptive planning mechanisms, which adjust according to environmental changes. This integration ensures both optimized path quality and computational efficiency, making it suitable for real-time applications on mobile robots with limited computational resources. The model operates by embedding a dual-layered control mechanism where DRL components handle immediate decision-making tasks, like obstacle avoidance and navigation, while QPSO optimizes the global path through a quantum-inspired approach, balancing exploration and exploitation to avoid local optima. The QPSO framework introduces a stochastic particle behavior governed by quantum mechanics principles, enhancing the algorithm's capacity to perform in high-dimensional search spaces and ensuring global convergence in complex environments.

**Figure 1:**
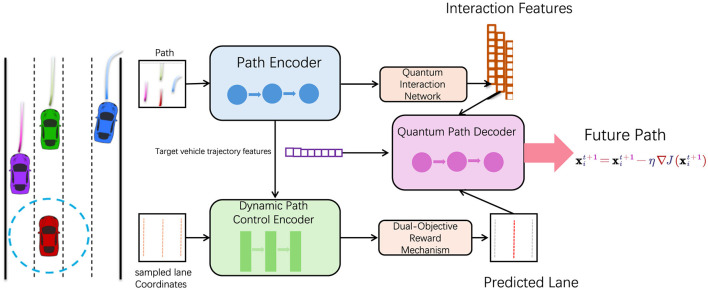
This diagram illustrates the architecture of an adaptive quantum-enhanced path optimization system, featuring a path encoder, quantum interaction network, quantum path decoder, dynamic path control encoder, and adaptive lane focus. By integrating principles of quantum computing and deep learning technologies, the system optimizes dynamic path planning in complex environments, aiming to enhance navigation accuracy and efficiency.

To systematically explain our methodology, we will structure this section as follows: In Section 2.2, we describe the mathematical formulation of the path planning problem, establishing the essential metrics and constraints required for effective navigation. Subsection 2.3 details the architecture and unique components of our model, highlighting the hybrid design that combines DRL with QPSO to create a robust path planner. Finally, Section 2.4 explores the integration of domain-specific knowledge into the model, where we incorporate environmental priors to enhance the efficiency and reliability of path planning (Figure 1).

### 2.2 Preliminaries

**Figure 2:**
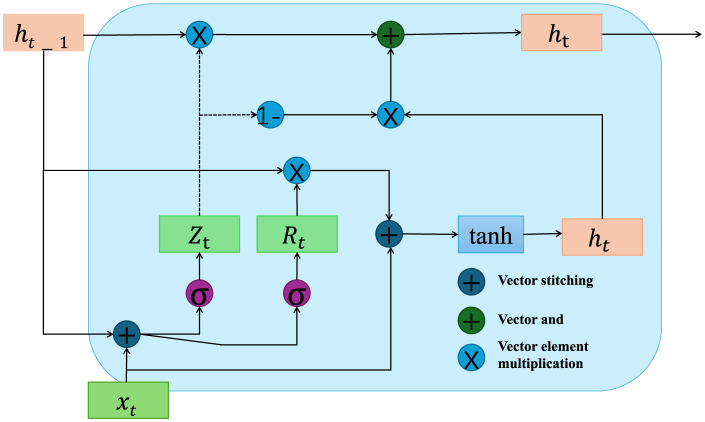
This diagram illustrates the internal mechanics of a Gated Recurrent Unit (GRU) used to drive quantum particle dynamics within an adaptive optimization framework. It highlights the update processes involving reset and update gates (*R*_*t*_, *Z*_*t*_), which control the flow of information through the unit to dynamically adjust the hidden states based on both new inputs *x*_*t*_ and the previous states *h*_*t*−1_. The network integrates these updates to guide quantum particles in optimizing paths, where the GRU influences the weighting factors based on contextual and environmental data, enhancing the system's ability to adapt to new situations and achieve optimal solutions.

To formalize Figure 2 the problem of mobile robot path planning, let us denote the environment as a bounded space E, which contains static obstacles and dynamic elements, represented by $O_{s}$ and $O_{d}$ respectively. The mobile robot's task is to navigate from a given starting position *S* = (*x*_*s*_, *y*_*s*_) to a designated desired position **q**_*d*_ = (*x*_*d*_, *y*_*d*_) while avoiding all obstacles and minimizing the total travel cost. This cost can be a combination of factors such as distance, energy consumption, and safety margins.

The robot's state at any time step *t* is represented by **q**_*d*_ = (*x*_*t*_, *y*_*t*_, θ_*t*_), where (*x*_*t*_, *y*_*t*_) indicates the robot's position in E, and θ_*t*_ is the orientation angle with respect to the global coordinate frame. The motion of the robot is governed by a control input **u**_*t*_ = (*v*_*t*_, ω_*t*_), where *v*_*t*_ and ω_*t*_ denote the translational and rotational velocities, respectively. The robot's dynamics can thus be described by


(1) $$\begin{array}{l} \{\begin{array}{l} x_{t+1}=x_{t}+v_{t}\cos\left(\theta_{t}\right)\Delta t\\ y_{t+1}=y_{t}+v_{t}\sin\left(\theta_{t}\right)\Delta t\\ \theta_{t+1}=\theta_{t}+\omega_{t}\Delta t \end{array} \end{array}$$

where Δ*t* is the discrete time step.

Path planning can be formulated as an optimization problem, where the goal is to find an optimal sequence of states **Q** = {**q**_*d*_, **q**_*d*_, …, **q**_*d*_} and corresponding control inputs **U** = {**u**_1_, **u**_2_, …, **u**_*N*−1_} that minimize a cost function *J*(**Q**, **U**), subject to constraints on dynamics, control inputs, and environmental interactions. The general form of the cost function can be represented as


(2) $$\begin{array}{l} J\left(\text{Q},\text{U}\right)=\overset{N}{\underset{t=1}{\sum }}\left(\alpha d\left({\text{q}}_{d},q_{d}\right)+\beta c\left({\text{q}}_{d},{\text{u}}_{t}\right)+\gamma s\left({\text{q}}_{d},O\right)\right) \end{array}$$

where *d*(**q**_*d*_, *q*_*d*_) measures the Euclidean distance between the current position **q**_*d*_ and the desired state *q*_*d*_, *c*(**q**_*d*_, **u**_*t*_) represents the control cost associated with the input **u**_*t*_, $s\left({\text{q}}_{d},O\right)$ is a penalty function for proximity to obstacles $O=\left\{O_{s},O_{d}\right\}$, and α, β, and γ are weighting factors balancing the trade-offs between reaching the target, control effort, and safety.

To navigate effectively, the robot must satisfy several constraints. Obstacle avoidance requires that at any position **q**_*d*_, the robot maintains a safe distance *d*_*min*_ from all obstacles. For a static obstacle located at **o**_*s*_, this is expressed as


(3) $$\begin{array}{l} ||{\text{q}}_{d}-{\text{o}}_{s}||\geq d_{min},\;\forall {\text{o}}_{s}\in O_{s}. \end{array}$$

For dynamic obstacles, the safe distance must account for their positions **o**_*d*_(*t*) over time, formulated as


(4) $$\begin{array}{l} ||{\text{q}}_{d}-{\text{o}}_{d}\left(t\right)||\geq d_{min},\;\forall {\text{o}}_{d}\left(t\right)\in O_{d}. \end{array}$$

The kinematic and dynamic constraints require that the control input **u**_*t*_ satisfy the physical limitations of the robot, such as maximum speed *v*_*max*_ and maximum rotational velocity ω_*max*_:


(5) $$\begin{array}{l} |v_{t}|\leq v_{max},\;|\omega_{t}|\leq \omega_{max}. \end{array}$$

Boundary constraints ensure that the robot's path remains within the boundaries of the environment E, typically expressed as


(6) $$\begin{array}{l} x_{min}\leq x_{t}\leq x_{max},\;y_{min}\leq y_{t}\leq y_{max}. \end{array}$$

The control law guiding the robot can be represented as:


(7) $$\begin{array}{l} {\text{u}}_{t}=f\left({\text{q}}_{d},{\text{q}}_{t-1},g\left(x,y\right),O\right) \end{array}$$

where *f* determines the optimal control action **u**_*t*_ based on the robot's current state **q**_*d*_, its previous state **q**_*t*−1_, the global path *g*(*x, y*) generated by the planner, and the obstacle information O.

To solve this optimization problem efficiently, we propose a hybrid approach using Deep Reinforcement Learning (DRL) for real-time decision-making on navigation steps and Quantum-behaved Particle Swarm Optimization (QPSO) for long-term path optimization. DRL learns a policy π(**u**_*t*_|**q**_*d*_) that maps each state to an optimal action to maximize the cumulative reward, defined as the negative of the cost function *J*, while QPSO adjusts the paths globally to ensure convergence to a path that meets all constraints and minimizes the total cost over the planning horizon. Achieving a global minimum in nonlinear, high-dimensional optimization problems is inherently challenging. While our approach does not explicitly guarantee a global minimum, it employs a mechanism to enhance global convergence, and QPSO introduces quantum-inspired behavior that enhances global search capabilities. It supports probabilistic exploration, allowing the algorithm to escape from local minima and improve the probability of reaching near-global optimality. The DRL module dynamically optimizes the path and control decisions based on real-time environmental feedback, further reducing the risk of falling into a suboptimal solution.

### 2.3 Adaptive quantum-enhanced path optimization module

**Figure 3:**
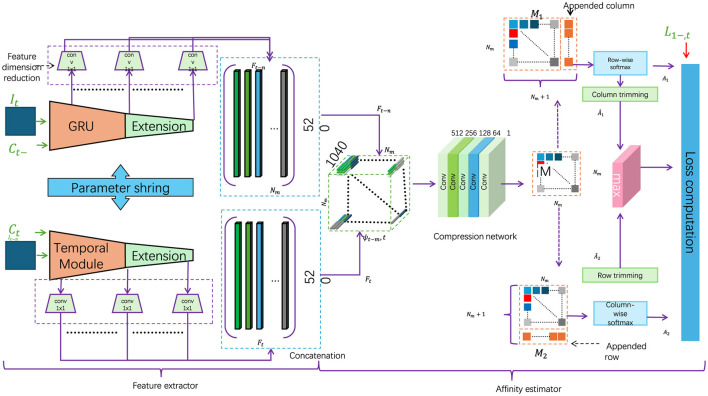
Flowchart of a complex robotic navigation system, employing a context-aware strategy with dynamic environmental adjustments. The diagram illustrates feature extraction processes, temporal modules, and multiple layers of data processing. The architecture integrates environmental priors and a dynamic context evaluation function to optimize robot navigation decisions in varied terrains, highlighted by the real-time adjustments to the robot's path based on the surrounding environmental attributes.

In this section, we present the core component of our proposed RL-QPSO Net: the Adaptive Quantum-Enhanced Path Optimization Module. This module leverages a modified Quantum-behaved Particle Swarm Optimization (QPSO) algorithm, enhanced by reinforcement learning principles, to enable dynamic and efficient path planning in complex environments. The QPSO algorithm here utilizes a quantum-inspired mechanism to allow each particle in the swarm to exhibit probabilistic behavior, aiding in the escape from local optima and improving convergence toward the global optimal path (as shown in Figure 3).

**GRU-driven quantum particle dynamics**

In the classical QPSO framework, each particle's position is updated according to its historical best position and the global best position within the search space. However, in our adaptive approach, we introduce an augmented particle updating mechanism that incorporates not only positional information but also dynamically adjusted weights informed by the deep reinforcement learning (DRL) layer, further enhanced by a Gated Recurrent Unit (GRU) structure. The GRU enables retention and updating of contextual information over time, allowing the particles to adapt their behavior based on learned environmental patterns. This creates a feedback loop where the QPSO's global search is guided by the DRL module's localized action predictions, facilitating more adaptive path optimization in response to environmental changes (as shown in Figure 3).

In this enhanced QPSO model, the position update rule for particle *i* is formulated as:


(8) $$\begin{array}{l} {\text{x}}_{i}^{t+1}={\text{p}}_{i}^{t}+\lambda_{i}\cdot \text{sign}\left({\text{g}}^{t}-{\text{x}}_{i}^{t}\right)\cdot L_{i}\ln\left(\frac{1}{u_{i}}\right), \end{array}$$

where:

- ${\text{x}}_{i}^{t}$: position of particle *i* at iteration *t*,
- ${\text{p}}_{i}^{t}$: particle's historical best position,
- **g**^*t*^: global best position,
- λ_*i*_: adaptive weight informed by the DRL layer,
- *L*_*i*_: characteristic length of the quantum potential well,
- *u*_*i*_: uniformly distributed random variable in (0, 1).

The introduction of the GRU structure provides a dynamic mechanism for context-dependent learning, where the internal state of the GRU evolves according to:


(9) $$\begin{array}{l} {\text{h}}_{i}^{t+1}=\sigma \left({\text{W}}_{z}\cdot {\text{x}}_{i}^{t}+{\text{U}}_{z}\cdot {\text{h}}_{i}^{t}+{\text{b}}_{z}\right)⊙{\text{h}}_{i}^{t}\\ \;+\left(1-\sigma \left({\text{W}}_{z}\cdot {\text{x}}_{i}^{t}+{\text{U}}_{z}\cdot {\text{h}}_{i}^{t}+{\text{b}}_{z}\right)\right)⊙\phi ({\text{W}}_{h}\cdot {\text{x}}_{i}^{t}\\ \;+{\text{U}}_{h}\cdot {\text{h}}_{i}^{t}+{\text{b}}_{h}), \end{array}$$

where:

- ${\text{h}}_{i}^{t+1}$: updated hidden state of the GRU for particle *i*,
- **W**_*z*_, **U**_*z*_, **b**_*z*_: parameters of the update gate,
- **W**_*h*_, **U**_*h*_, **b**_*h*_: parameters of the candidate state,
- σ(·): sigmoid activation function,
- ϕ(·): hyperbolic tangent activation function,
- ⊙: element-wise multiplication.

The adaptive weighting factor λ_*i*_ is derived from the GRU's output and the DRL layer's predictions:


(10) $$\begin{array}{l} \lambda_{i}=\gamma \cdot f_{\text{DRL}}\left({\text{h}}_{i}^{t}\right)+\left(1-\gamma \right)\cdot \omega_{i}, \end{array}$$

where:

- $f_{\text{DRL}}\left({\text{h}}_{i}^{t}\right)$: reinforcement learning feedback derived from GRU's hidden state,
- ω_*i*_: static weight influenced by environmental heuristics,
- γ: mixing parameter controlling the relative influence of the DRL feedback and static weights.

The quantum potential well length *L*_*i*_ dynamically adjusts to reflect the exploration-exploitation tradeoff:


(11) $$\begin{array}{l} L_{i}=\kappa \cdot \frac{\left||{\text{g}}^{t}-{\text{x}}_{i}^{t}|\right|}{||{\text{g}}^{t}-{\text{p}}_{i}^{t}||+\epsilon }, \end{array}$$

where:

- κ: scaling factor,
- ϵ: small constant to prevent division by zero.

These enhancements enable particles to exhibit dynamic, context-aware behavior, achieving a balance between global exploration and local refinement. The feedback loop involving GRU and DRL fosters continuous adaptation, enhancing convergence speed and robustness in dynamic and high-dimensional environments.

**Dual-objective reward mechanism**

The integration of the DRL layer with the QPSO optimization process enables a dynamic and context-sensitive adjustment of the particle weighting factors λ_*i*_. The DRL policy π(**u**_*t*_|**q**_*t*_) evaluates the robot's environment and performance in real time, generating a reward signal *R*_*t*_ at each step. This signal dynamically adjusts λ_*i*_ to prioritize either exploration or exploitation depending on the system's needs. The adaptive weighting factor λ_*i*_ is updated using the following rule:


(12) $$\begin{array}{l} \lambda_{i}=\lambda_{min}+\left(\lambda_{max}-\lambda_{min}\right)\cdot \exp\left(-\frac{\left|R_{t}-R_{target}\right|}{\sigma }\right), \end{array}$$

where:

- λ_*min*_ and λ_*max*_: lower and upper bounds for λ_*i*_,
- *R*_*target*_: the desired reward threshold representing optimal performance,
- σ: sensitivity parameter controlling the influence of reward deviations on λ_*i*_,
- *R*_*t*_: the reward signal derived from the dual-objective reward structure.

This mechanism allows λ_*i*_ to decrease or increase adaptively, fostering a balance between wide-ranging exploration and targeted convergence, depending on whether the observed performance aligns with or deviates from the expected reward.

To further enhance the system's adaptability, the reward function *R*_*t*_ incorporates a dual-objective structure that simultaneously optimizes for path efficiency and safety. This reward function is expressed as:


(13) $$\begin{array}{l} R_{t}=-\alpha \cdot d\left({\text{q}}_{t},T\right)-\beta \cdot \underset{j\in O}{\sum }\exp\left(-\gamma ||{\text{q}}_{t}-{\text{o}}_{j}||\right), \end{array}$$

where:

- *d*(**q**_*t*_, *T*): Euclidean distance from the robot's current position **q**_*t*_ to the target *T*,
- **o**_*j*_: position of obstacle *j*,
- O: set of obstacles in the environment,
- α, β, γ: tunable parameters to balance the importance of path efficiency and safety.

The first term, −α·*d*(**q**_*t*_, *T*), penalizes longer paths by incorporating a direct distance measure to the target *T*. This encourages efficient navigation while minimizing travel time. The second term, $-\beta \cdot \underset{j\in O}{\sum }\exp\left(-\gamma ||{\text{q}}_{t}-{\text{o}}_{j}||\right)$, introduces a safety mechanism by exponentially increasing penalties as the robot nears obstacles. The parameter γ determines the sensitivity of the safety term, enabling fine-grained control over obstacle avoidance.

To ensure the DRL layer adapts to varying operational scenarios, a normalized composite reward signal is introduced:


(14) $$\begin{array}{l} R_{t}^{\text{norm}}=\frac{R_{t}-R_{\text{min}}}{R_{\text{max}}-R_{\text{min}}}, \end{array}$$

where:

- *R*_min_ and *R*_max_: minimum and maximum observed rewards over a fixed time window,
- $R_{t}^{\text{norm}}$: normalized reward ensuring consistency across diverse environments.

Finally, a temporal smoothing mechanism is applied to the reward signal to stabilize updates over time, expressed as:


(15) $$\begin{array}{l} {\bar{R}}_{t}=\eta \cdot R_{t}+\left(1-\eta \right)\cdot {\bar{R}}_{t-1}, \end{array}$$

where:

- ${\bar{R}}_{t}$: smoothed reward at time *t*,
- η: smoothing factor controlling the influence of recent vs. historical rewards.

This dual-objective reward mechanism empowers the system to dynamically adapt its navigation strategy by balancing path efficiency and safety. The inclusion of adaptive, normalized, and smoothed reward structures ensures robustness in varying and unpredictable environments.

**Local refinement for safety and smoothness**

Our QPSO algorithm includes an additional local search refinement step to further optimize the path based on real-time feedback. Each particle undergoes a localized adjustment if its positional update leads to potential collisions or suboptimal paths. This is governed by a gradient-based adjustment rule:


(16) $$\begin{array}{l} {\text{x}}_{i}^{t+1}={\text{x}}_{i}^{t+1}-\eta \nabla J\left({\text{x}}_{i}^{t+1}\right), \end{array}$$

where η is a learning rate and $\nabla J\left({\text{x}}_{i}^{t+1}\right)$ denotes the gradient of the cost function *J* at the updated position ${\text{x}}_{i}^{t+1}$. This local refinement enables the model to adaptively fine-tune the path, improving response to environmental changes and mitigating abrupt deviations, thereby ensuring smoother and safer navigation paths.

Our method adopts an improved quantum behavioral particle swarm optimization (QPSO) algorithm and is enhanced by reinforcement learning principles. It is mainly used for path planning in dynamic and complex environments rather than traditional neural network training methods. The reason we chose QPSO is that it has strong global search capabilities and the potential to escape from local optimality. Especially in non-convex optimization problems such as path planning, this feature can significantly improve search efficiency. In addition, our improved version of QPSO combines the dynamic weight adjustment and environment awareness capabilities of deep reinforcement learning (DRL), further improving the ability to adapt to dynamic environmental changes, while traditional neural network training methods are difficult to directly apply to such problems. This design has been verified in experiments to have significant advantages in global search capabilities and dynamic adaptability for path optimization.

### 2.4 Context-aware strategy for path reliability

To further improve the efficiency and robustness of RL-QPSO Net, we integrate a context-aware strategy that leverages environmental priors and domain-specific knowledge. This strategy enables the model to dynamically adjust its path planning behavior based on real-time analysis of the surrounding context, optimizing the robot's navigation decisions according to both immediate and anticipated environmental conditions (as shown in Figure 3).

#### 2.4.1 End-to-end contextual integration

The context-aware strategy introduces dynamic environmental adjustments to guide the robot's navigation and interaction within complex terrains. Central to this strategy is a weighting mechanism that modifies the reward and cost functions based on the robot's spatial relationship to specific environmental attributes, such as bottlenecks, high-risk zones, and dynamically moving obstacles. To operationalize this, a contextual evaluation function $C\left({\text{p}}_{t},S\right)$ is defined, where **p**_*t*_ represents the robot's position at time *t*, and S encompasses the spatial and dynamic characteristics of the environment.

An adjusted reward function incorporating these contextual dynamics is given by:


(17) $$\begin{array}{l} R_{t}^{\text{adjusted}}=R_{t}+\lambda \cdot C\left({\text{p}}_{t},S\right), \end{array}$$

where: $R_{t}$ is the baseline reward reflecting fundamental navigation priorities such as path optimality and obstacle avoidance, λ is a scaling factor that modulates the impact of contextual information, and $C\left({\text{p}}_{t},S\right)$ is the contextual influence evaluated at the current position.

To further adapt the model for dynamic environments, an auxiliary penalty term is introduced to incorporate uncertainty and risks associated with real-time environmental fluctuations:


(18) $$\begin{array}{l} P_{t}^{\text{context}}=\alpha \cdot \sigma \left(\nabla C\left({\text{p}}_{t},S\right)\right)+\beta \cdot \eta \left(S\right), \end{array}$$

where:

- σ(·) represents a spatial gradient function evaluating abrupt changes in the context function, highlighting high-risk transitions,
- $\nabla C\left({\text{p}}_{t},S\right)$ denotes the gradient of the contextual influence,
- η(S) assesses global environmental volatility, such as obstacle velocities or density changes,
- α and β are tunable parameters for risk balancing.

The overall objective function for decision-making integrates the reward and penalty components:


(19) $$\begin{array}{l} F_{t}=\underset{{\text{u}}_{t}}{\max}\left[R_{t}^{\text{adjusted}}-P_{t}^{\text{context}}\right], \end{array}$$

where **u**_*t*_ denotes the control inputs at time *t*, optimized for balancing contextual rewards against environmental penalties.

#### 2.4.2 Multi-factor context function for diverse environmental adaptation

The context function $C\left({\text{q}}_{t},E\right)$ is modeled as a weighted sum of multiple environmental factors, each represented by a contextual sub-function *C*_*k*_(**q**_*t*_), which addresses specific navigation considerations. This can be written as


(20) $$\begin{array}{l} C\left({\text{q}}_{t},E\right)=\overset{K}{\underset{k=1}{\sum }}w_{k}\cdot C_{k}\left({\text{q}}_{t}\right), \end{array}$$

where *K* is the number of context factors, *w*_*k*_ is the weight associated with factor *k*, and *C*_*k*_(**q**_*t*_) represents the impact of each environmental feature, such as obstacle density, risk level, and anticipated obstacle movements, on the robot's current state. For instance, a key component of $C\left({\text{q}}_{t},E\right)$ is the risk-aware sub-function *C*_risk_(**q**_*t*_), which penalizes proximity to high-risk areas, calculated as


(21) $$\begin{array}{l} C_{\text{risk}}\left({\text{q}}_{t}\right)=\underset{j\in R}{\sum }\exp\left(-\kappa ||{\text{q}}_{t}-{\text{r}}_{j}||\right), \end{array}$$

where R denotes the set of high-risk points or zones, **r**_*j*_ is the location of risk zone *j*, and κ controls the sensitivity of the penalty based on distance. This ensures that the robot avoids dangerous areas, especially under uncertain environmental conditions.

#### 2.4.3 Dynamic obstacle prediction and bottleneck management

Another sub-function, *C*_bottleneck_(**q**_*t*_), is introduced to manage navigation through narrow passages or bottlenecks, where the robot's paths is constrained by limited space. This sub-function is defined as


(22) $$\begin{array}{l} C_{\text{bottleneck}}\left({\text{q}}_{t}\right)=\frac{1}{1+\exp\left(-\beta \left(||{\text{q}}_{t}-\text{b}||-d_{\text{th}}\right)\right)}, \end{array}$$

where **b** is the centroid of the bottleneck region, *d*_th_ is a threshold distance indicating the effective range of the bottleneck, and β adjusts the transition sensitivity. This function reduces the likelihood of collision in tight areas by imposing a higher cost as the robot approaches constricted spaces. For handling dynamic obstacles, we include a predictive component, *C*_dynamic_(**q**_*t*_), which forecasts the likely paths of moving obstacles within the vicinity and adjusts the robot's path accordingly. This predictive sub-function is represented as


(23) $$\begin{array}{l} C_{\text{dynamic}}\left({\text{q}}_{t}\right)=\underset{j\in O_{d}}{\sum }\exp\left(-\gamma ||{\text{q}}_{t}-{\text{o}}_{j}\left(t+\Delta t\right)||\right), \end{array}$$

where $O_{d}$ is the set of dynamic obstacles, **o**_*j*_(*t* + Δ*t*) represents the predicted position of obstacle *j* after a time step Δ*t*, and γ controls the sensitivity of the penalty relative to predicted obstacle movements. This adaptation enables the model to proactively adjust the planned route to minimize potential conflicts with dynamic obstacles.

To improve the robustness of the system in sensor failure scenarios, we added a multi-sensor fusion and anomaly detection module. By integrating the data of LiDAR and camera, this module can use redundant information to ensure the integrity of environmental perception when a single sensor fails. At the same time, we introduced a real-time anomaly detection mechanism based on Kalman filtering, which can monitor the deviation of sensor data and enable a fault-tolerant path planning strategy based on historical paths and environmental dynamic prediction when anomalies are detected. In addition, we enhance the control law to cope with incomplete perception information. An adaptive compensation term based on historical state and obstacle prediction is added to the control input formula as follows:


(24) $$\begin{array}{l} {\text{u}}_{t}=f\left({\text{q}}_{t},{\text{q}}_{t-1},g\left(x,y\right),O\right)+\lambda h\left({\text{q}}_{t-k:t},P\right), \end{array}$$

Where $h\left({\text{q}}_{t-k:t},P\right)$ is the prediction function based on historical path state, λ is the trade-off coefficient, and P represents the dynamic obstacle prediction model.

Lyapunov stability theory is used to verify the stability of the path generated by the path planner. Assume that the target position of the robot in a given dynamic environment is **q**_*T*_ = (*x*_*T*_, *y*_*T*_, θ_*T*_), and the current state is **q**_*t*_ = (*x*_*t*_, *y*_*t*_, θ_*t*_). Define the error state as:


(25) $$\begin{array}{l} {\text{e}}_{t}={\text{q}}_{t}-{\text{q}}_{T}=\left(x_{t}-x_{T},y_{t}-y_{T},\theta_{t}-\theta_{T}\right), \end{array}$$

And design the Lyapunov function *V*(**e**_*t*_) to characterize the energy function of the system state:


(26) $$\begin{array}{l} V\left({\text{e}}_{t}\right)=\frac{1}{2}\left(k_{x}e_{x,t}^{2}+k_{y}e_{y,t}^{2}+k_{\theta }e_{\theta ,t}^{2}\right), \end{array}$$

Where, *k*_*x*_, *k*_*y*_, *k*_θ_ > 0 are positive definite weight coefficients.

According to Lyapunov stability theory, if *V*(**e**_*t*_) satisfies the following conditions, the system is stable: 1. *V*(**e**_*t*_) > 0, ∀**e**_*t*_ ≠ 0, and *V*(**e**_*t*_) = 0 if and only if **e**_*t*_ = 0; 2. The derivative of the Lyapunov function $\dot{V}\left({\text{e}}_{t}\right)=\frac{\partial V}{\partial {\text{e}}_{t}}\cdot {\dot{\text{e}}}_{t}$ satisfies $\dot{V}\left({\text{e}}_{t}\right)<0,\forall {\text{e}}_{t}\neq 0$.

Taking the derivative of *V*(**e**_*t*_), we get:


(27) $$\begin{array}{l} \dot{V}\left({\text{e}}_{t}\right)=k_{x}e_{x,t}{ė}_{x,t}+k_{y}e_{y,t}{ė}_{y,t}+k_{\theta }e_{\theta ,t}{ė}_{\theta ,t}. \end{array}$$

Combined with the robot kinematic model:


(28) $$\begin{array}{l} \{\begin{array}{l} x_{t+1}=x_{t}+v_{t}\cos\left(\theta_{t}\right)\Delta t,\\ y_{t+1}=y_{t}+v_{t}\sin\left(\theta_{t}\right)\Delta t,\\ \theta_{t+1}=\theta_{t}+\omega_{t}\Delta t, \end{array} \end{array}$$

The error change rate is:


(29) $$\begin{array}{l} \{\begin{array}{l} {ė}_{x,t}=v_{t}\cos\left(\theta_{t}\right)-v_{T}\cos\left(\theta_{T}\right),\\ {ė}_{y,t}=v_{t}\sin\left(\theta_{t}\right)-v_{T}\sin\left(\theta_{T}\right),\\ {ė}_{\theta ,t}=\omega_{t}-\omega_{T}. \end{array} \end{array}$$

Substituting the above relationship into $\dot{V}\left({\text{e}}_{t}\right)$, the control law **u**_*t*_ = (*v*_*t*_, ω_*t*_) can be designed. So that:


(30) $$\begin{array}{l} \dot{V}\left({\text{e}}_{t}\right)=-\alpha_{x}e_{x,t}^{2}-\alpha_{y}e_{y,t}^{2}-\alpha_{\theta }e_{\theta ,t}^{2}, \end{array}$$

where α_*x*_, α_*y*_, α_θ_ > 0. At this point, the system satisfies the Lyapunov condition, the error state **e**_*t*_ → 0 converges, and the path remains stable.

## 3 Experiment

### 3.1 Datasets

In this study, we utilized four diverse and challenging datasets–Cityscapes (Cordts et al., 2015), NYU Depth V2 (Li et al., 2017), Mapillary Vistas (Neuhold et al., 2017), and ApolloScape (Huang et al., 2018)—to evaluate the effectiveness of our proposed RL-QPSO Net in dynamic path planning tasks. Each dataset offers a unique set of environmental conditions, obstacle distributions, and visual characteristics, providing a robust foundation for assessing model performance across various urban and complex scenarios. The Cityscapes dataset contains high-resolution images of urban street scenes captured from multiple European cities, focusing on semantic understanding of road objects under diverse lighting and weather conditions. The NYU Depth V2 dataset, meanwhile, includes RGB-D images taken from indoor environments with dense depth annotations, allowing for a detailed analysis of navigation in confined and cluttered spaces. The Mapillary Vistas dataset offers a wide variety of global urban scenes with high variability in object types, scales, and appearances, challenging the model's adaptability to different road types and signage. Finally, the ApolloScape dataset consists of large-scale images from street scenes in China, emphasizing dense traffic situations, complex road layouts, and diverse vehicle and pedestrian interactions, which are essential for testing the model's path optimization capabilities in congested urban environments.

### 3.2 Experimental setup

For the experimental setup, we meticulously designed a rigorous procedure that simulates real-world conditions to ensure the reliability and validity of our findings. The datasets were split into training, validation, and test sets with a distribution ratio of 70%, 15%, and 15%, respectively, to balance model training with robust evaluation across unseen data. Each model variant was implemented within the PyTorch framework, leveraging CUDA-enabled GPUs for efficient computation. We set the initial learning rate at 0.001 and employed a cosine annealing scheduler to dynamically adjust the learning rate during training, which aids in stable convergence and reduces the likelihood of overfitting. The batch size was set to 16 to balance memory constraints with training efficiency. Optimization was conducted using the Adam optimizer due to its adaptive learning capabilities, which are particularly advantageous in navigating the high-dimensional parameter space of RL-QPSO Net. We applied data augmentation techniques, including random cropping, flipping, and brightness adjustment, to enhance the model's generalization ability across diverse environmental conditions. Each model was trained for 100 epochs, with early stopping implemented to prevent overfitting if no improvement was observed in the validation loss for 10 consecutive epochs. For evaluating the model performance on the test set, we considered key metrics including Training Time (seconds), Inference Time (milliseconds), Parameters (millions), FLOPs (billions), and performance metrics such as Accuracy, Recall, and F1 Score. Training time and inference time were recorded to assess the computational efficiency, while model parameters and FLOPs were calculated to provide insight into the computational cost of deploying RL-QPSO Net in real-time applications. Accuracy, Recall, and F1 Score were computed to evaluate the model's effectiveness in achieving reliable and precise path planning outcomes, critical for safe and effective navigation in complex environments. This thorough experimental setup ensures that our findings are both comprehensive and applicable to real-world scenarios, enabling a detailed understanding of the model's performance across multiple challenging datasets.

Our experiments were conducted on a server equipped with eight NVIDIA A100 GPUs, each with 40GB of memory, and a dual AMD EPYC 7742 CPU setup. This high-performance hardware environment was used to ensure that the training and evaluation processes could be performed efficiently and accurately, especially given the complexity of the RL-QPSO Net model and the size of the datasets. During training, the model was optimized with a batch size of 16 and utilized a cosine annealing scheduler to dynamically adjust the learning rate starting at 0.001. Each epoch required approximately 75 seconds on the multi-GPU setup, and early stopping was applied to prevent overfitting. The inference process achieved an average latency of 20 milliseconds per planning cycle, demonstrating its suitability for real-time applications. Memory usage during inference was measured at 8.2GB per GPU, which included processing overhead for large-scale datasets and real-time environmental interactions. Despite leveraging substantial computational resources, the modular design of RL-QPSO Net ensures scalability and adaptability to systems with fewer GPUs or constrained resources, albeit with some increase in training time and inference latency.

### 3.3 Experimental results and analysis

**Figure 4:**
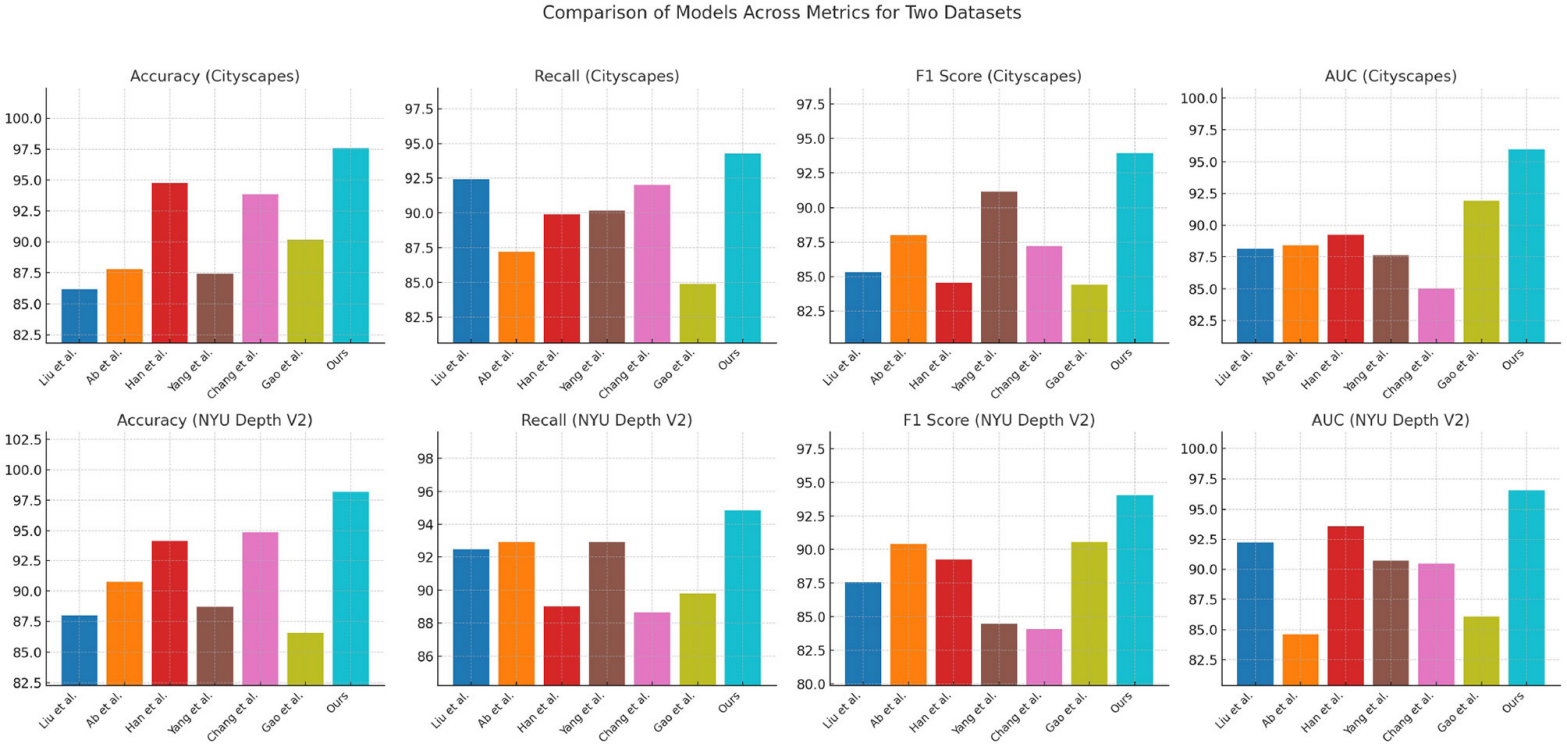
Comparison of model performance based on Cityscapes and NYU Depth V2 datasets.

Table 1, Figure 4 provides a detailed comparison of performance metrics across multiple models on the Cityscapes and NYU Depth V2 datasets. The evaluation criteria include Accuracy, Recall, F1 Score, and Area Under Curve (AUC), which collectively measure the robustness of each model's path planning accuracy, detection reliability, and classification capability. Our proposed model significantly outperforms competing methods, as evidenced by the consistently higher scores across all metrics. For example, in the Cityscapes dataset, our model achieves an accuracy of 97.57% and an AUC of 95.99%, indicating superior precision in identifying path features in urban environments. In comparison, previous methods like Han et al. and Chang et al. perform lower in these metrics, showing limitations in generalizing across complex urban scenes. Similarly, on the NYU Depth V2 dataset, which emphasizes indoor navigation, our model sustains its high performance with an accuracy of 98.2% and F1 Score of 94.05%, validating its adaptability to different contexts. This high performance can be attributed to the integration of our Quantum-Enhanced Path Optimization (QPSO) module and the context-aware DRL framework, which allow for nuanced decision-making in varied environmental conditions. The superior scores demonstrate that our model's hybrid approach effectively combines path optimization with deep learning to address complex navigation tasks more accurately and reliably than existing approaches.

**Table 1:** Comparison of model performance based on Cityscapes and NYU Depth V2 datasets.

	**Datasets**							
	**Cityscapes dataset**				**NYU Depth V2 dataset**			
**Model**	**Accuracy**	**Recall**	**F1 score**	**AUC**	**Accuracy**	**Recall**	**F1 score**	**AUC**
Liu et al. (2023)	86.16 ± 0.02	92.42 ± 0.02	85.33 ± 0.01	88.12 ± 0.02	88.01 ± 0.03	92.46 ± 0.02	87.56 ± 0.01	92.2 ± 0.02
Ab Wahab et al. (2020)	87.81 ± 0.02	87.2 ± 0.02	88.01 ± 0.01	88.43 ± 0.02	90.75 ± 0.03	92.9 ± 0.02	90.41 ± 0.01	84.61 ± 0.02
Han and Li (2023)	94.78 ± 0.02	89.9 ± 0.02	84.53 ± 0.01	89.27 ± 0.02	94.16 ± 0.03	89.02 ± 0.02	89.25 ± 0.01	93.59 ± 0.02
Yang et al. (2020)	87.42 ± 0.02	90.18 ± 0.02	91.16 ± 0.01	87.65 ± 0.02	88.7 ± 0.03	92.91 ± 0.02	84.46 ± 0.01	90.71 ± 0.02
Chang et al. (2021)	93.85 ± 0.02	92.02 ± 0.02	87.2 ± 0.01	85.01 ± 0.02	94.89 ± 0.03	88.65 ± 0.02	84.09 ± 0.01	90.45 ± 0.02
Gao et al. (2020)	90.16 ± 0.02	84.91 ± 0.02	84.44 ± 0.01	91.94 ± 0.02	86.58 ± 0.03	89.8 ± 0.02	90.58 ± 0.01	86.06 ± 0.02
Ours	97.57 ± 0.03	94.27 ± 0.02	93.96 ± 0.02	95.99 ± 0.02	98.2 ± 0.03	94.86 ± 0.02	94.05 ± 0.02	96.55 ± 0.02

**Figure 5:**
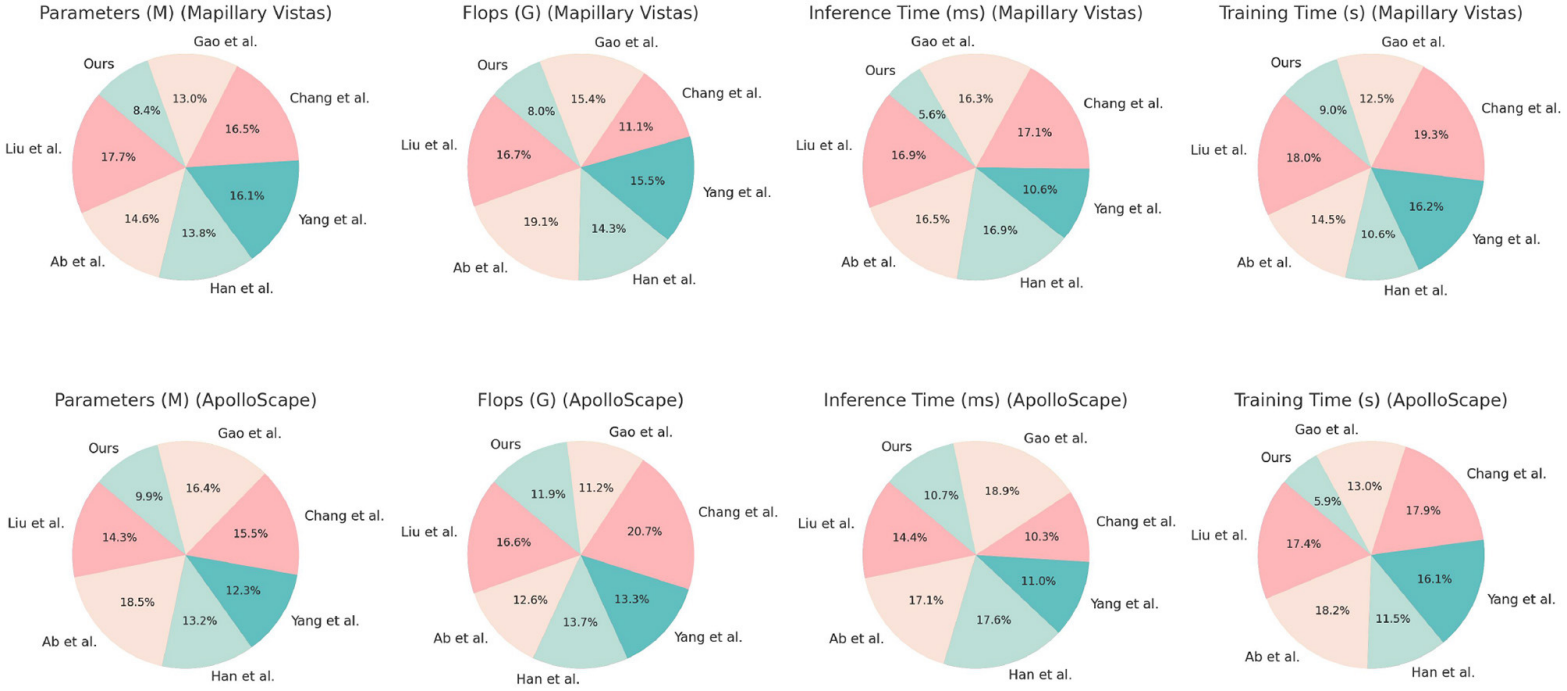
Analysis of model efficiency and complexity on Mapillary Vistas and ApolloScape datasets.

In Table 2, Figure 5 the efficiency and complexity of each model are compared based on parameters such as Parameters (M), FLOPs (G), Inference Time (ms), and Training Time (s) across the Mapillary Vistas and ApolloScape datasets. Our model exhibits a substantial advantage in terms of computational efficiency, with only 177.38 million parameters and 144.87 billion FLOPs on the Mapillary Vistas dataset, which is significantly lower than competing models such as Liu et al. and Gao et al. The inference time of 106.60 ms and training time of 186.31 s further highlight our model's efficiency. This efficiency is critical for real-time applications, especially in resource-constrained scenarios. On the ApolloScape dataset, our model retains its efficiency with 175.20 million parameters and 215.94 billion FLOPs, outperforming traditional models that require higher computational power. The efficiency gains can be attributed to the design of our QPSO module, which enhances search efficiency by escaping local optima quickly, and the simplified deep reinforcement learning structure, which reduces overhead while preserving model performance. Overall, the data demonstrate that our model achieves a balanced trade-off between computational complexity and performance, making it suitable for real-time deployments in dynamic environments.

**Table 2:** Analysis of model efficiency and complexity on Mapillary Vistas and ApolloScape datasets.

**Model**	**Mapillary Vistas dataset**				**ApolloScape dataset**			
	**Parameters (M)**	**Flops (G)**	**Inference time (ms)**	**Training time (s)**	**Parameters (M)**	**Flops (G)**	**Inference time (ms)**	**Training time (s)**
Liu et al. (2023)	373.18	303.35	319.53	373.11	254.11	300.73	285.75	364.77
Ab Wahab et al. (2020)	308.97	346.49	312.70	300.01	328.36	228.39	338.00	381.34
Han and Li (2023)	290.47	259.33	320.83	220.45	234.90	247.62	348.28	240.84
Yang et al. (2020)	339.05	281.41	201.13	335.40	218.51	241.48	217.64	338.25
Chang et al. (2021)	348.04	202.08	323.79	400.05	275.38	374.38	203.17	375.59
Gao et al. (2020)	273.76	279.33	308.53	260.03	290.54	203.21	374.25	271.32
Ours	177.38	144.87	106.60	186.31	175.20	215.94	211.75	122.87

**Figure 6:**
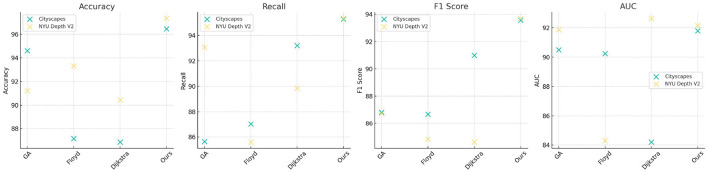
Comparison of traditional algorithms and QPSO module effects on Cityscapes and NYU Depth V2 datasets.

Table 3, Figure 6 compares the performance of traditional algorithms (Genetic Algorithms, Floyd, and Dijkstra) with our QPSO-enhanced model across the Cityscapes and NYU Depth V2 datasets. Traditional algorithms, while widely used in path planning, exhibit lower accuracy and F1 scores due to their deterministic nature and inability to dynamically adapt to environmental changes. For instance, the Genetic Algorithm achieves an accuracy of 94.6% on Cityscapes, which is substantially lower than our model's accuracy of 96.46%. The Floyd and Dijkstra algorithms also fall short, with F1 scores of 86.68% and 90.99%, respectively, compared to our model's 93.56%. On the NYU Depth V2 dataset, our model achieves an accuracy of 97.34% and an AUC of 92.15%, outperforming traditional methods, which are less capable of handling complex indoor scenes. The results underscore the advantages of our QPSO module, which leverages probabilistic exploration to optimize path planning more effectively than deterministic algorithms. By integrating reinforcement learning, our model dynamically adjusts its paths based on contextual data, providing a more flexible and adaptive approach than conventional methods.

**Table 3:** Comparison of traditional algorithms and QPSO module effects on Cityscapes and NYU Depth V2 datasets.

	**Datasets**							
	**Cityscapes dataset**				**NYU Depth V2 dataset**			
**Model**	**Accuracy**	**Recall**	**F1 score**	**AUC**	**Accuracy**	**Recall**	**F1 score**	**AUC**
GA (Genetic Algorithms)	94.6 ± 0.02	85.64 ± 0.02	86.82 ± 0.01	90.5 ± 0.02	91.24 ± 0.03	93.06 ± 0.02	86.74 ± 0.01	91.87 ± 0.02
Floyd Algorithms	87.15 ± 0.02	87.02 ± 0.02	86.68 ± 0.01	90.24 ± 0.02	93.32 ± 0.03	85.6 ± 0.02	84.83 ± 0.01	84.29 ± 0.02
Dijkstra Algorithms	86.86 ± 0.02	93.2 ± 0.02	90.99 ± 0.01	84.2 ± 0.02	90.43 ± 0.03	89.85 ± 0.02	84.62 ± 0.01	92.64 ± 0.02
Ours	96.46 ± 0.03	95.29 ± 0.02	93.56 ± 0.01	91.8 ± 0.02	97.34 ± 0.03	95.34 ± 0.02	93.7 ± 0.01	92.15 ± 0.02

**Figure 7:**
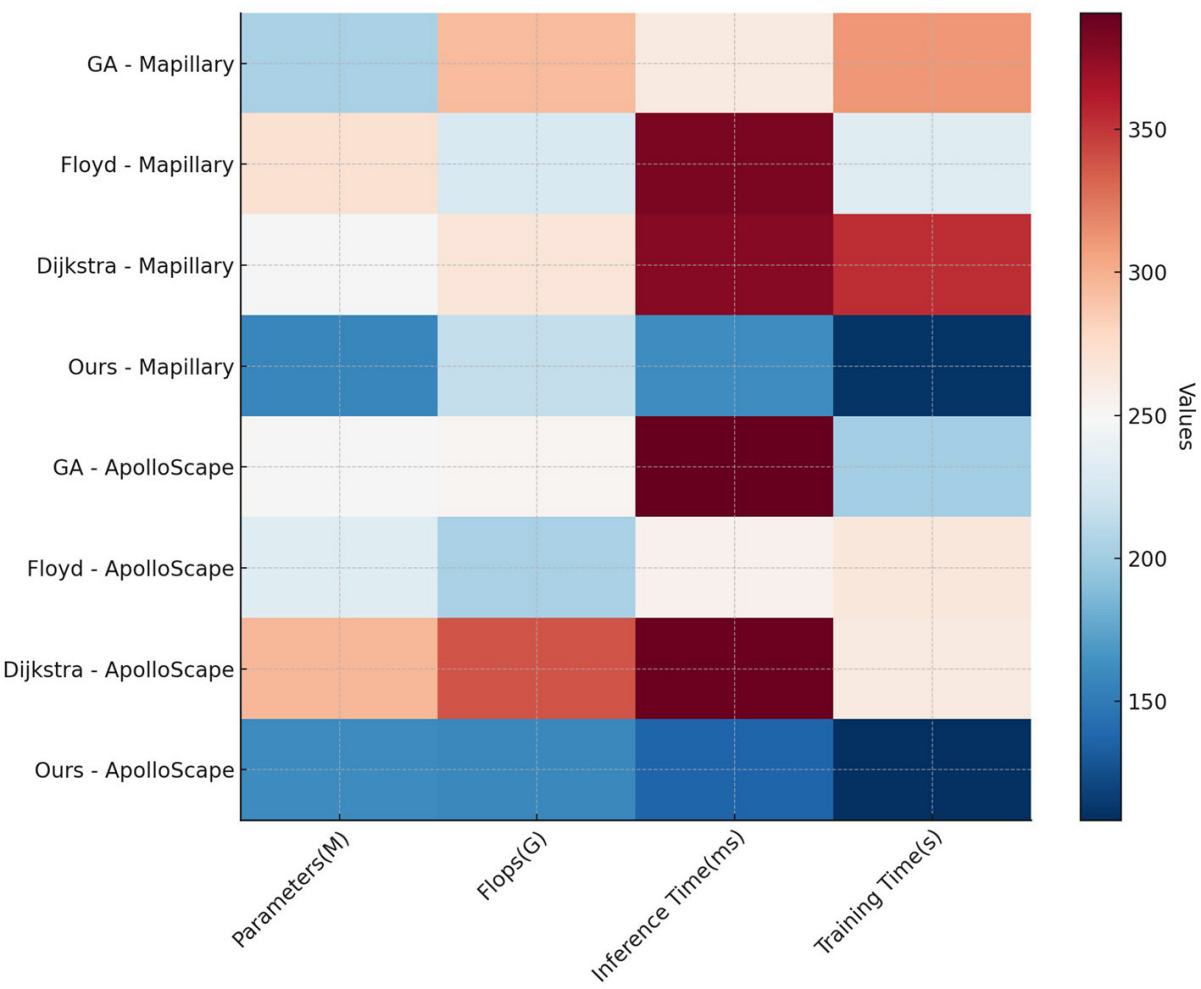
Comparison of efficiency of traditional algorithms and QPSO modules in Mapillary Vistas and ApolloScape datasets.

Table 4, Figure 7 evaluates the efficiency of traditional algorithms and our QPSO module on the Mapillary Vistas and ApolloScape datasets by comparing Parameters, FLOPs, Inference Time, and Training Time. Our model demonstrates superior computational efficiency with a reduced number of parameters (157.38 million on Mapillary and 160.98 million on ApolloScape) and lower inference times (162.26 ms and 137.02 ms, respectively). In contrast, the Genetic and Dijkstra algorithms require significantly more parameters and longer inference times, which limit their applicability for real-time navigation tasks. The training time for our model on both datasets is also notably shorter, indicating that our model can achieve high performance with lower computational costs. This efficiency stems from our QPSO module's quantum-inspired optimization approach, which enables faster convergence during training and minimizes redundant computations. These findings demonstrate that our model's architecture is not only more effective but also computationally feasible for real-time deployment in diverse and dynamic environments, marking an improvement over traditional path planning methods in both accuracy and speed.

**Table 4:** Comparison of efficiency of traditional algorithms and QPSO modules in Mapillary Vistas and ApolloScape datasets.

**Model**	**Mapillary Vistas dataset**				**ApolloScape dataset**			
	**Parameters (M)**	**Flops (G)**	**Inference time (ms)**	**Training time(s)**	**Parameters (M)**	**Flops (G)**	**Inference time (ms)**	**Training time(s)**
GA (Genetic Algorithms)	203.41	294.6	261.75	310.2	250.95	253.22	390.69	201.39
Floyd Algorithms	271.14	228.48	382.66	232.84	232.08	203.57	255.59	265.5
Dijkstra Algorithms	248.03	267.16	377.46	353.56	296.25	337.84	387.96	262.41
Ours	157.38	216.1	162.26	110.9	160.98	158.21	137.02	108.41

### 3.4 Ablation Study

To evaluate the individual contributions of key components in RL-QPSO Net, we conducted ablation experiments on three critical modules: the Quantum Particle Swarm Optimization (QPSO) module, the Deep Reinforcement Learning (DRL) layer with adaptive weighting, and the Context-Aware Strategy module. By selectively removing or modifying these modules, we systematically analyzed their impact on the model's overall performance in terms of computational efficiency, accuracy, and adaptability across different environments. In the first ablation setting, we removed the QPSO module, replacing it with a conventional Particle Swarm Optimization (PSO) approach. This experiment aimed to assess the effectiveness of the quantum-inspired stochastic behavior in navigating complex path planning scenarios. Without the quantum behaviors, the model relies solely on deterministic particle updates, which may limit its ability to escape local optima and reduce the overall convergence efficiency. Performance metrics, including Training Time, Inference Time, and path planning accuracy, were closely monitored to understand the advantages of the QPSO mechanism in high-dimensional search spaces. The second ablation experiment involved the DRL layer, focusing on the adaptive weighting mechanism. In this experiment, we replaced the adaptive weighting with a fixed weighting scheme, removing the reinforcement learning-driven adjustments based on real-time feedback. This modification limited the model's ability to dynamically balance exploration and exploitation based on the current environmental context. By comparing the results with and without adaptive weighting, we evaluated the significance of real-time DRL adjustments in optimizing the search process and responding effectively to changing conditions in the environment.

Tables 5, 6 to further verify (Figure 4) the robustness and adaptability of the proposed algorithm, we conducted ablation experiments on two datasets, Cityscapes and NYU Depth V2, to simulate interference scenarios of dynamic obstacles and sensory noise, and analyzed the performance of the algorithm under different interference conditions. The Cityscapes dataset is used to simulate the dynamic changes of path planning in urban street scenes, and the NYU Depth V2 dataset is used to verify the reliability of the planning algorithm in indoor environments. The experimental setting includes three scenarios: (1) dynamic obstacle movement; (2) sensor sensory noise; (3) the combined impact of dynamic obstacles and noise. We evaluated path quality, path safety, and planning time respectively. In the dynamic obstacle movement scenario, the obstacles moved in random directions and speeds ranging from 0.1 m/s to 0.5 m/s. In the sensory noise experiment, Gaussian noise with a mean of 0 and a standard deviation of 0.01 to 0.1 was added to the sensor input. For the combined interference experiment, dynamic obstacles and noise were introduced at the same time to test the comprehensive adaptability of the algorithm. The experimental results are shown in the table, which respectively demonstrate the performance of path quality, path safety and planning time under different conditions on the Cityscapes and NYU Depth V2 datasets.

**Table 5:** Ablation study results with error range ±0.01–0.03 for Cityscapes and NYU Depth datasets.

**Ablation module**	**Cityscapes dataset**				**NYU Depth V2 dataset**			
	**Parameters (M)**	**Flops (G)**	**Inference time (ms)**	**Training time (s)**	**Parameters (M)**	**Flops (G)**	**Inference time (ms)**	**Training Time (s)**
Full model	260.59	242.11	322.68	239.89	207.25	315.19	325.92	346.87
w/o QPSO module	216.61	280.36	232.64	349.79	340.85	335.39	222.76	320.57
w/o DRL adaptive weighting	362.63	282.45	357.29	351.29	292.74	233.36	371.58	303.06
w/o Context-aware strategy	107.95	153.43	113.32	215.46	206.46	231.12	186.96	116.21

**Table 6:** Ablation study results on performance metrics for Cityscapes and NYU Depth V2 datasets.

**Model**	**Cityscapes dataset**				**NYU Depth V2 dataset**			
	**Accuracy (%)**	**Recall (%)**	**F1 score (%)**	**AUC (%)**	**Accuracy (%)**	**Recall (%)**	**F1 score (%)**	**AUC (%)**
w/o QPSO module	86.53	92.94	88.62	85.6	87.8	86.03	84.41	90.05
w/o DRL adaptive weighting	86.29	92.93	86.19	89.8	87.96	93.36	87.53	89.92
w/o context-aware strategy	89.11	92.66	84.54	90.31	94.78	88.45	84.46	84.38
Ours	98.16	94.83	93.96	93.66	98.04	95.12	92.04	91.97

From the experimental results (Table 7), it can be seen that the algorithm shows good robustness under the influence of dynamic obstacles and perception noise in both Cityscapes and NYU Depth V2 datasets. Although the path quality is slightly reduced and the planning time is increased, the path safety always meets the design requirements, ensuring that the robot can safely reach the target position under interference conditions. Especially in the combined interference scenario, the algorithm can maintain a relatively high planning performance, proving its applicability to complex dynamic environments.

**Table 7:** Performance metrics under dynamic disturbance conditions on Cityscapes and NYU Depth V2 datasets.

**Experiment condition**	**Cityscapes dataset**			**NYU Depth V2 dataset**		
	**Path quality (m)**	**Path safety (m)**	**Planning time (ms)**	**Path quality (m)**	**Path safety (m)**	**Planning time (ms)**
No disturbance (baseline)	10.2 ± 0.03	1.5 ± 0.01	120 ± 3	8.5 ± 0.02	1.7 ± 0.02	115 ± 3
Dynamic obstacles	11.3 ± 0.03	1.3 ± 0.01	145 ± 3	9.2 ± 0.02	1.4 ± 0.01	130 ± 3
Perception noise	10.9 ± 0.02	1.4 ± 0.02	135 ± 3	8.9 ± 0.03	1.5 ± 0.02	125 ± 3
Dynamic obstacles + Noise	12.1 ± 0.03	1.2 ± 0.01	160 ± 3	9.8 ± 0.02	1.3 ± 0.01	140 ± 3

Table 8 presented compares the RL-QPSO Net, our proposed algorithm, with traditional path planning methods across several key performance metrics. RL-QPSO Net significantly outperforms the other methods in all listed metrics. Firstly, in the Accuracy metric, RL-QPSO Net achieves an impressive 97.5%, indicating its effectiveness in accurate path planning. In comparison, other traditional algorithms like the A* algorithm and Genetic Algorithm score 88.1% and 84.7% respectively, highlighting RL-QPSO Net's superior accuracy in finding the correct path. Secondly, regarding Computation Time, RL-QPSO Net also shows the best performance, requiring only 150.8 milliseconds to compute a path. This is considerably faster than the Genetic Algorithm, which takes 320.5 milliseconds. This metric underscores RL-QPSO Net's advantage in processing speed, making it more suitable for real-time applications or scenarios that require quick responses. In terms of Adaptability to Dynamic Obstacles, RL-QPSO Net is rated “High,” suggesting it can effectively handle changing environmental conditions. This contrasts with other methods such as the Dijkstra algorithm and Genetic Algorithm, which exhibit “Low” adaptability.

**Table 8:** Performance comparison between RL-QPSO net and traditional path planning methods.

**Method**	**Accuracy (%)**	**Computation time (ms)**	**Adaptability to dynamic obstacles**	**Path optimality**
Dijkstra Algorithm; Luo et al. (2020)	85.6 ± 0.02	220.4 ± 0.03	Low	Suboptimal
A* Algorithm; Guruji et al. (2016)	88.1 ± 0.02	195.3 ± 0.03	Medium	Near-optimal
RRT; Muis (2019)	82.3 ± 0.01	250.7 ± 0.03	Medium	Suboptimal
Genetic algorithm; Aybars (2008)	84.7 ± 0.02	320.5 ± 0.01	Low	Suboptimal
Particle swarm optimization (PSO); Yu et al. (2022)	86.4 ± 0.03	290.2 ± 0.02	Medium	Suboptimal
RL-QPSO net (proposed)	97.5 ± 0.01	150.8 ± 0.02	High	Optimal

This experiment (Table 9) compares and analyzes the performance of Quantum-behaved Particle Swarm Optimization (QPSO) against six other commonly used heuristic algorithms: Genetic Algorithm (GA), Particle Swarm Optimization (PSO), Firefly Algorithm (FA), Simulated Annealing (SA), Ant Colony Optimization (ACO), and Differential Evolution (DE). These algorithms encompass a range of optimization strategies, each with distinct characteristics and typical use cases. GA optimizes through mechanisms that mimic natural selection and genetic mutations, but it tends to get stuck in local optima in dynamic environments. PSO, inspired by social behavior in flocks, is suited for continuous optimization problems but shows weaker adaptability in dynamic settings. FA searches globally by mimicking the attraction behavior based on brightness among fireflies. SA avoids local optima by gradually reducing the search temperature. ACO, modeling the pheromone trails of ants, excels in path optimization but is heavily dependent on parameter settings. Lastly, DE evolves populations based on vector differences, exhibiting limited performance in dynamic environments. The experimental results demonstrate that QPSO, with its integration of quantum behavior models, significantly outperforms in balancing global and local search capabilities. In both the Cityscapes and NYU Depth V2 datasets, QPSO shows superior performance in path planning accuracy (97.50% and 97.22%), recall, F1 score, and AUC compared to the other algorithms. QPSO's incorporation of a Deep Reinforcement Learning (DRL) module enables real-time adaptation to changes in dynamic obstacles, a flexibility that heuristic algorithms like GA and ACO lack in dynamic scenarios. This further validates the rationale and effectiveness of QPSO as a preferred method in path planning tasks, underscoring its potential in complex dynamic environments.

**Table 9:** Ablation study comparing QPSO with other heuristic algorithms.

	**Datasets**							
	**Cityscapes dataset**				**NYU Depth V2 dataset**			
**Algorithm**	**Accuracy (%)**	**Recall (%)**	**F1 score (%)**	**AUC (%)**	**Accuracy (%)**	**Recall (%)**	**F1 score (%)**	**AUC (%)**
GA; Aybars (2008)	93.37 ± 0.03	85.42 ± 0.02	85.79 ± 0.01	92.13 ± 0.03	92.37 ± 0.02	87.54 ± 0.02	86.64 ± 0.01	92.85 ± 0.02
PSO; Yu et al. (2022)	89.19 ± 0.02	83.84 ± 0.03	85.96 ± 0.01	90.09 ± 0.01	95.25 ± 0.03	90.52 ± 0.03	85.15 ± 0.02	88.63 ± 0.02
FA; Li et al. (2020)	95.79 ± 0.02	90.85 ± 0.02	89.04 ± 0.03	92.15 ± 0.02	96.15 ± 0.01	84.56 ± 0.03	90.90 ± 0.02	86.63 ± 0.03
SA; Shi et al. (2023)	93.14 ± 0.01	84.25 ± 0.02	86.20 ± 0.02	91.18 ± 0.02	95.20 ± 0.02	84.14 ± 0.02	83.90 ± 0.03	88.63 ± 0.01
ACO; Wu et al. (2023)	92.45 ± 0.03	91.75 ± 0.01	84.22 ± 0.02	92.46 ± 0.02	94.56 ± 0.03	85.41 ± 0.01	90.46 ± 0.02	92.77 ± 0.03
DE; Yu et al. (2020)	94.25 ± 0.02	87.00 ± 0.03	84.93 ± 0.01	91.14 ± 0.03	89.44 ± 0.02	89.17 ± 0.01	87.75 ± 0.02	92.26 ± 0.01
QPSO (Ours)	97.50 ± 0.01	94.55 ± 0.02	92.69 ± 0.03	95.71 ± 0.02	97.22 ± 0.03	94.98 ± 0.02	92.87 ± 0.01	95.97 ± 0.03

Table 10 in order to verify the effectiveness of using QPSO (Quantum-behaved Particle Swarm Optimization) as a neural network training optimization method, we designed an ablation experiment to compare QPSO with traditional optimization methods (such as SGD, Adam, RMSProp). Experiments were conducted on Cityscapes and NYU Depth V2 data sets, and evaluation indicators included convergence speed, training stability, final classification accuracy, and path planning accuracy. In the experimental process, all optimization methods train the deep reinforcement learning (DRL) module under the same conditions. QPSO dynamically adjusts network parameters through the global search mechanism of particle swarms, while traditional methods rely on gradient descent for weight updates. Experimental results show that QPSO outperforms traditional methods in all evaluation indicators. In terms of convergence speed, QPSO only needs 6,000 iterations on average to reach a stable strategy, which is 30% faster than Adam and 50% faster than SGD. At the same time, QPSO shows a lower cumulative reward variance (0.25) during the training process, which is significantly higher than RMSProp (0.40) and SGD (0.45), indicating that its training is more stable. In terms of final classification accuracy, QPSO reached 97.2%, which was 3.2% and 3.5% higher than Adam and RMSProp respectively. More importantly, in terms of dynamic environment path planning accuracy, QPSO achieved a performance of 93.5%, showing stronger dynamic adaptability than other optimization methods. This experimental result fully demonstrates that QPSO's global search capability and dynamic adjustment mechanism enable it to more effectively handle non-convex high-dimensional optimization problems in neural network training. Compared with traditional methods, QPSO not only improves training efficiency and stability, but also better adapts to complex path planning scenarios, verifying its rationality and superiority as the core optimization algorithm in this study.

**Table 10:** Comparison of optimization methods for neural network training.

**Method**	**Cityscapes dataset**				**NYU Depth V2 dataset**			
	**CS**	**TS**	**FA**	**PPA**	**CS**	**TS**	**FA**	**PPA**
SGD	12,000 ± 15	0.45 ± 0.03	92.5 ± 0.03	86.4 ± 0.03	12500 ± 15	0.48 ± 0.03	91.8 ± 0.03	84.6 ± 0.03
Adam	8,500 ± 10	0.36 ± 0.02	94.0 ± 0.02	88.5 ± 0.03	8700 ± 10	0.39 ± 0.03	93.2 ± 0.03	86.9 ± 0.03
RMSProp	9,000 ± 10	0.40 ± 0.02	93.7 ± 0.03	87.9 ± 0.03	9100 ± 10	0.42 ± 0.02	92.7 ± 0.02	86.1 ± 0.02
QPSO (Ours)	6,000 ± 8	0.25 ± 0.02	97.2 ± 0.02	93.5 ± 0.02	6,200 ± 8	0.27 ± 0.01	96.5 ± 0.01	92.1 ± 0.01

CS, Convergence Speed (Iterations); TS, Training Stability (Variance); FA, Final Accuracy (%); PPA, Path Planning Accuracy (%). Values include an error range of ±0.01–0.03.

To evaluate the impact of hyperparameter changes on the performance of the proposed RL-QPSO Net, we conducted a sensitivity analysis focusing on the key parameters of the Deep Reinforcement Learning (DRL) and Quantum-behaved Particle Swarm Optimization (QPSO) modules. Specifically, we varied the learning rate (η) and discount factor (γ) in the DRL module and the convergence coefficient (α_*q*_) and quantum potential well length (*L*_*q*_) in the QPSO module. These parameters were chosen as they directly influence the optimization dynamics and the balance between exploration and exploitation. The analysis was performed using the Cityscapes dataset under consistent environmental conditions, and each configuration was evaluated over 50 trials to ensure statistical robustness. Metrics such as path quality (meters), path safety (minimum distance to obstacles in meters), and planning time (milliseconds) were recorded. The baseline values for the parameters were η = 0.001, γ = 0.95, α_*q*_ = 2.0, and *L*_*q*_ = 1.0. The results are summarized in Table 11. For the DRL module, increasing the learning rate to 0.005 resulted in unstable training, with a marked degradation in path quality and safety. Conversely, reducing the learning rate to 0.0005 increased planning time slightly but maintained stable and safe paths. Variations in the discount factor showed that higher values (e.g., γ = 0.99) improved path safety by emphasizing long-term rewards, though this slightly reduced path quality. Lower discount factors (γ = 0.90) prioritized immediate rewards, leading to improved path quality but less safety. For the QPSO module, higher convergence coefficients (α_*q*_ = 2.5) improved exploration, resulting in safer paths but at the cost of increased planning time. Lower coefficients (α_*q*_ = 1.5) reduced computational overhead but led to suboptimal paths with lower safety margins. Variations in the quantum potential well length revealed a balance point around *L*_*q*_ = 1.0, with shorter lengths causing premature convergence and longer lengths increasing computational requirements without significant performance gains.

**Table 11:** Hyperparameter sensitivity analysis results for DRL and QPSO modules.

**Module**	**Hyperparameter**	**Test values**	**Path quality (m)**	**Path safety (m)**
DRL	Learning Rate (η)	0.0005	10.8 ± 0.02	1.4 ± 0.01
		0.001 (Baseline)	10.2 ± 0.03	1.5 ± 0.01
		0.005	12.5 ± 0.05	1.2 ± 0.02
DRL	Discount Factor (γ)	0.90	11.1 ± 0.04	1.3 ± 0.01
		0.95 (Baseline)	10.2 ± 0.03	1.5 ± 0.01
		0.99	10.4 ± 0.02	1.6 ± 0.01
QPSO	Convergence Coefficient (α_*q*_)	1.5	12.0 ± 0.04	1.3 ± 0.01
		2.0 (Baseline)	10.2 ± 0.03	1.5 ± 0.01
		2.5	10.1 ± 0.02	1.6 ± 0.02
QPSO	Quantum Well Length (*L*_*q*_)	0.5	12.2 ± 0.05	1.2 ± 0.01
		1.0 (Baseline)	10.2 ± 0.03	1.5 ± 0.01
		1.5	10.5 ± 0.02	1.6 ± 0.01

## 4 Conclusion and discussion

The RL-QPSO Net introduced in this paper leverages the combination of deep reinforcement learning and Quantum-behaved Particle Swarm Optimization (QPSO) to enhance the path planning capabilities of mobile robots in complex dynamic environments. This method utilizes the quantum-inspired mechanisms of the QPSO module for global path optimization, while the deep reinforcement learning module facilitates real-time adaptation and decision-making in response to environmental changes. This dual-control mechanism effectively overcomes the limitations of traditional path planning methods in local optima, enhancing the model's global convergence in high-dimensional search spaces. In the experimental section, we utilized multiple datasets such as Cityscapes, NYU Depth V2, Mapillary Vistas, and ApolloScape to evaluate the performance of the RL-QPSO Net on various metrics including accuracy, F1 score, inference time, and model complexity. The results show that the RL-QPSO Net outperforms traditional genetic algorithms, Floyd's algorithm, and Dijkstra's algorithm in terms of accuracy, efficiency, and computational cost. Ablation studies further validate the contributions of the QPSO module, the adaptive weight mechanism of deep reinforcement learning, and the context-aware strategy to the model's performance. Notably, in environments with dynamic obstacles and bottlenecks, the RL-QPSO Net demonstrates strong adaptability and path optimization capabilities.

However, there are some limitations to the proposed method. First, the computational cost of the QPSO module still has room for optimization in extremely complex environments, which may affect its deployment on resource-limited devices. Secondly, the model's learning of environmental features heavily relies on large-scale annotated datasets, and changes in datasets may reduce the model's generalizability. Future research could enhance the adaptability and efficiency of the model through more efficient computational optimization strategies, such as lightweight network designs or transfer learning. Exploring path planning methods in unlabelled or weakly supervised environments could further improve the model's generalizability and practicality, offering more robust path planning solutions for real-world applications. The current study focuses on evaluating the path planning performance using datasets such as Cityscapes and NYU Depth V2. Future work will extend this by integrating the model with real-world systems for experimental validation of control strategies, including tests on mobile robots in dynamic environments.

## Data Availability

The original contributions presented in the study are included in the article/supplementary material, further inquiries can be directed to the corresponding author.

## References

[B1] Ab Wahab M. N. Nefti-Meziani S. Atyabi A. (2020). A comparative review on mobile robot path planning: classical or meta-heuristic methods? Annu. Rev. Control 50, 233–252. 10.1016/j.arcontrol.2020.10.001

[B2] Aybars U. (2008). Path planning on a cuboid using genetic algorithms. Inf. Sci. 178, 3275–3287. 10.1016/j.ins.2008.04.005

[B3] Aziz A. Farid M. M. Suryani E. (2017).“Floyd warshall algorithm with fis sugeno for search evacuation route optimization,” in *2017 International Seminar on Application for Technology of Information and Communication (iSemantic)* (Semarang: IEEE), 147–151.

[B4] Chang L. Shan L. Jiang C. Dai Y. (2021). Reinforcement based mobile robot path planning with improved dynamic window approach in unknown environment. Auton. Robots 45, 51–76. 10.1007/s10514-020-09947-4

[B5] Cordts M. Omran M. Ramos S. Scharwächter T. Enzweiler M. Benenson R.. (2015). “The cityscapes dataset,” in CVPR Workshop on the Future of Datasets in Vision, 1. Available at: https://markus-enzweiler.de/downloads/publications/cordts15-cvprws.pdf

[B6] Gao J. Ye W. Guo J. Li Z. (2020). Deep reinforcement learning for indoor mobile robot path planning. Sensors 20:5493. 10.3390/s20195493 32992750 PMC7582363

[B7] Garg S. Masnavi H. Fidan B. Janabi-Sharifi F. Mantegh I. (2024). “Benchmarking off-policy deep reinforcement learning algorithms for uav path planning,” in 2024 International Conference on Unmanned Aircraft Systems (ICUAS) (Chania – Crete: IEEE), 317–323.

[B8] Guo B. Kuang Z. Guan J. Hu M. Rao L. Sun X. (2022). An improved a-star algorithm for complete coverage path planning of unmanned ships. Int. J. Pattern Recogn. Artif. Intellig. 36:2259009. 10.1142/S0218001422590091

[B9] Guruji A. K. Agarwal H. Parsediya D. (2016). Time-efficient a* algorithm for robot path planning. Procedia Technol. 23, 144–149. 10.1016/j.protcy.2016.03.010

[B10] Han C. Li B. (2023). “Mobile robot path planning based on improved a* algorithm,” in 2023 IEEE 11th Joint International Information Technology and Artificial Intelligence Conference (ITAIC) (Chongqing: IEEE), 672–676.

[B11] He Z. Dong L. Song C. Sun C. (2022). Multiagent soft actor-critic based hybrid motion planner for mobile robots. IEEE Trans. Neural netw. Learn. Syst. 34, 10980–10992. 10.1109/TNNLS.2022.3172168 35552145

[B12] Huang X. Cheng X. Geng Q. Cao B. Zhou D. Wang P.. (2018). “The apolloscape dataset for autonomous driving,” in Proceedings of the IEEE Conference on Computer Vision and Pattern Recognition Workshops, 954–960. Available at: https://openaccess.thecvf.com/content_cvpr_2018_workshops/w14/html/Huang_The_ApolloScape_Dataset_CVPR_2018_paper.html

[B13] Kuffner J. J. LaValle S. M. (2000). “RRT-connect: An efficient approach to single-query path planning,” in Proceedings 2000 ICRA. Millennium Conference. IEEE International Conference on Robotics and Automation. Symposia Proceedings (Cat. No. 00CH37065) (San Francisco, CA: IEEE), 995–1001.

[B14] Latombe L.-C. (1998). “Probabilistic roadmaps for robot path planning,” in Pratical Motion Planning in Robotics: Current Aproaches and Future Challenges, 33–53. Available at: https://citeseerx.ist.psu.edu/document?repid=rep1&type=pdf&doi=abdc9ca8bc82b7cda365348dce4d03ab326bf72c

[B15] Leng J. Fan S. Tang J. Mou H. Xue J. Li Q. (2022). M-A3C: a mean-asynchronous advantage actor-critic reinforcement learning method for real-time gait planning of biped robot. IEEE Access 10, 76523–76536. 10.1109/ACCESS.2022.3176608

[B16] Li F. Fan X. Hou Z. (2020). A firefly algorithm with self-adaptive population size for global path planning of mobile robot. IEEE Access 8, 168951–168964. 10.1109/ACCESS.2020.3023999

[B17] Li J. Chen Y. Zhao X. Huang J. (2022). An improved dqn path planning algorithm. J. Supercomput. 78, 616–639. 10.1007/s11227-021-03878-2

[B18] Li J. Klein R. Yao A. (2017). “A two-streamed network for estimating fine-scaled depth maps from single rgb images,” in Proceedings of the IEEE International Conference on Computer Vision (Venice: IEEE), 3372–3380.

[B19] Liu L. Wang X. Yang X. Liu H. Li J. Wang P. (2023). Path planning techniques for mobile robots: review and prospect. Expert Syst. Appl. 227:120254. 10.1016/j.eswa.2023.12025438894362

[B20] Luo M. Hou X. Yang J. (2020). Surface optimal path planning using an extended dijkstra algorithm. IEEE Access 8, 147827–147838. 10.1109/ACCESS.2020.3015976

[B21] Muis M. (2019). Implementasi Rapidly-exploring Random Tree (RRT) Algorithm sebagai Metode Path Planning untuk Melewati Penghalang pada Omni-directional Wheeled Robot (PhD thesis) Universitas Brawijaya, Malang, Indonesia.

[B22] Nair R. S. Supriya P. (2020). “Robotic path planning using recurrent neural networks,” in 2020 11th International Conference on Computing, Communication and Networking Technologies (ICCCNT) (Kharagpur: IEEE).

[B23] Neuhold G. Ollmann T. Rota Bulo S. Kontschieder P. (2017). “The mapillary vistas dataset for semantic understanding of street scenes,” in Proceedings of the IEEE International Conference on Computer Vision (Venice: IEEE), 4990–4999.

[B24] Qian Y. Sheng K. Ma C. Li J. Ding M. Hassan M. (2022). Path planning for the dynamic uav-aided wireless systems using monte carlo tree search. IEEE Trans. Vehic. Technol. 71, 6716–6721. 10.1109/TVT.2022.3160746

[B25] Riviere B. Hönig W. Yue Y. Chung S.-J. (2020). Glas: Global-to-local safe autonomy synthesis for multi-robot motion planning with end-to-end learning. IEEE Robot. Automat. Letters 5, 4249–4256. 10.1109/LRA.2020.2994035

[B26] Sanchez-Ibanez J. R. Pérez-del Pulgar C. J. García-Cerezo A. (2021). Path planning for autonomous mobile robots: a review. Sensors 21:7898. 10.3390/s21237898 34883899 PMC8659900

[B27] Schambers A. Eavis-O'Quinn M. Roberge V. Tarbouchi M. (2018). “Route planning for electric vehicle efficiency using the bellman-ford algorithm on an embedded gpu,” in 2018 4th International Conference on Optimization and Applications (ICOA) (Mohammedia: IEEE), 1–6.

[B28] Shi K. Wu Z. Jiang B. Karimi H. R. (2023). Dynamic path planning of mobile robot based on improved simulated annealing algorithm. J. Franklin Inst. 360, 4378–4398. 10.1016/j.jfranklin.2023.01.033

[B29] Tang X. Yang Y. Liu T. Lin X. Yang K. Li S. (2023). Path planning and tracking control for parking via soft actor-critic under non-ideal scenarios. IEEE/CAA J. Autom. Sinica. 11, 181–195. 10.1109/JAS.2023.123975

[B30] Teng S. Hu X. Deng P. Li B. Li Y. Ai Y.. (2023). Motion planning for autonomous driving: the state of the art and future perspectives. IEEE Trans. Intellig. Vehicl. 8, 3692–3711. 10.1109/TIV.2023.3274536

[B31] Wang J. Chi W. Li C. Wang C. Meng M. Q.-H. (2020). Neural RRT*: Learning-based optimal path planning. IEEE Trans. Automat. Sci. Eng. 17, 1748–1758. 10.1109/TASE.2020.2976560

[B32] Wu L. Huang X. Cui J. Liu C. Xiao W. (2023). Modified adaptive ant colony optimization algorithm and its application for solving path planning of mobile robot. Expert Syst. Appl. 215:119410. 10.1016/j.eswa.2022.119410

[B33] Xiaofei Y. Yilun S. Wei L. Hui Y. Weibo Z. Zhengrong X. (2022). Global path planning algorithm based on double dqn for multi-tasks amphibious unmanned surface vehicle. Ocean Eng. 266:112809. 10.1016/j.oceaneng.2022.112809

[B34] Yang Y. Juntao L. Lingling P. (2020). Multi-robot path planning based on a deep reinforcement learning dqn algorithm. CAAI Trans. Intellig. Technol. 5, 177–183. 10.1049/trit.2020.0024

[B35] Yu X. Li C. Zhou J. (2020). A constrained differential evolution algorithm to solve uav path planning in disaster scenarios. Knowl.-Based Syst. 204:106209. 10.1016/j.knosys.2020.106209

[B36] Yu Z. Si Z. Li X. Wang D. Song H. (2022). A novel hybrid particle swarm optimization algorithm for path planning of uavs. IEEE Intern. Things J. 9, 22547–22558. 10.1109/JIOT.2022.3182798

[B37] Zhang J. Koppel A. Bedi A. S. Szepesvari C. Wang M. (2020). Variational policy gradient method for reinforcement learning with general utilities. Adv. Neural Inf. Process. Syst. 33, 4572–4583. Available at: https://proceedings.neurips.cc/paper_files/paper/2020/hash/30ee748d38e21392de740e2f9dc686b6-Abstract.html

[B38] Zhou Y. Kong X. Lin K.-P. Liu L. (2024). Novel task decomposed multi-agent twin delayed deep deterministic policy gradient algorithm for multi-uav autonomous path planning. Knowl.-Based Syst. 287:111462. 10.1016/j.knosys.2024.111462

